# Why Testing Protocols Matter in Electrochemical Methane Oxidation: Insights from IrO_*x*_ in Acid

**DOI:** 10.1021/acsenergylett.5c01848

**Published:** 2025-09-08

**Authors:** José Alejandro Arminio-Ravelo, Silvia Favero, María Escudero-Escribano

**Affiliations:** † Department of Chemistry, 4321 University of Copenhagen, Universitetsparken 5, 2100 Copenhagen, Denmark; ‡ Catalan Institute of Nanoscience and Nanotechnology (ICN2), Edifici ICN2, CSIC and Barcelona Institute of Science and Technology, UAB Campus, 08193 Bellaterra, Barcelona, Spain; § Catalan Institution for Research and Advanced Studies (ICREA), Passeig de Lluís Companys, 23, 08010 Barcelona, Spain

Natural gas is often a byproduct of oil extraction, and when recovery of this gas is uneconomical, it is vented or flared, releasing methane and carbon dioxide in the atmosphere. In the past 15 years, 3.5% of all natural gas production has been flared yearly.^1^ Not only is methane a stronger greenhouse gas than CO_2_, with its emissions constituting the second largest cause of global warming, but the flaring of methane is also a futile waste of resources.^1^ Methane is the key reactant in the production of methanol, with a global production amount of 110 million tonnes in 2024.^2^ Moreover, methanol demand has been steadily increasing and is expected to grow further, driven by the demand for sustainable fuels, especially in maritime transportation. Currently, methanol is produced industrially via a two-step process involving the oxidation of methane to synthesis gas (syngas), a mixture of CO and H_2_,^3^ followed by its catalytic reduction to methanol. While this process is well-established, methane to methanol plants require high energy consumptions and are economically justified only at large-scale productions of methanol when generating at least 2500 t per day.^4^ The possibility of converting methane directly to methanol at small to middle-scale, and ideally at ambient temperature and pressure conditions would open a great opportunity to reduce the CO_2_ emissions, energy consumptions, and safety concerns of methanol production.^4^ This could be achieved by the electrochemical partial oxidation of methane, which would operate at low temperature, at ambient pressure and utilizing renewable electricity. Thus, electrochemical methods are highly attractive to convert methane into green methanol conversion in an efficient, sustainable, and decentralized way. However, this process remains a great challenge due to the difficulty of activating methane and selectively oxidizing it to methanol.^4,5^

Electrochemical activation of methane can proceed via direct or indirect activation.^6^ Direct methane activation involves the direct adsorption of methane on the electrode surface, as illustrated in Figure 1. In contrast, in the indirect approach, highly reactive species such as radicals are generated and can then activate methane in the vicinity of the electrochemical interface or in the bulk of the solution.^6^ The activation of the inert C–H bonds of methane is often the limiting factor in achieving high reaction rates.^4–8^ Furthermore, methanol is more reactive than methane, making CO_2_ the most favorable product. While the methane oxidation potential is 0.63 V vs RHE, the thermodynamic potential for methanol oxidation to CO_2_ is only 0.05 V vs RHE. As a result, once methane is activated, the greatest challenge becomes to stabilize the intermediates that favor the formation of methanol, avoiding the full oxidation to CO_2_. To this aim, various strategies have been employed for the direct electrochemical activation of methane on metal oxides, with the goal of enhancing the selectivity toward alcohol production.^9–13^ Given the complexity of electrochemical methane oxidation, several experimental parameters are likely to influence the activity and selectivity of metal oxides. However, the effect of these parameters remains largely overlooked, and systematic analysis of their effect is lacking. Moreover, the scattered experimental results reported in the literature have been obtained under vastly different conditions, making them difficult to compare. To address this gap, in this work, we aim to investigate the effect of three key experimental parameters: temperature, surface area estimation, and electrochemical technique (Figure 1), on the electrochemical methane oxidation.

**Figure 1:**
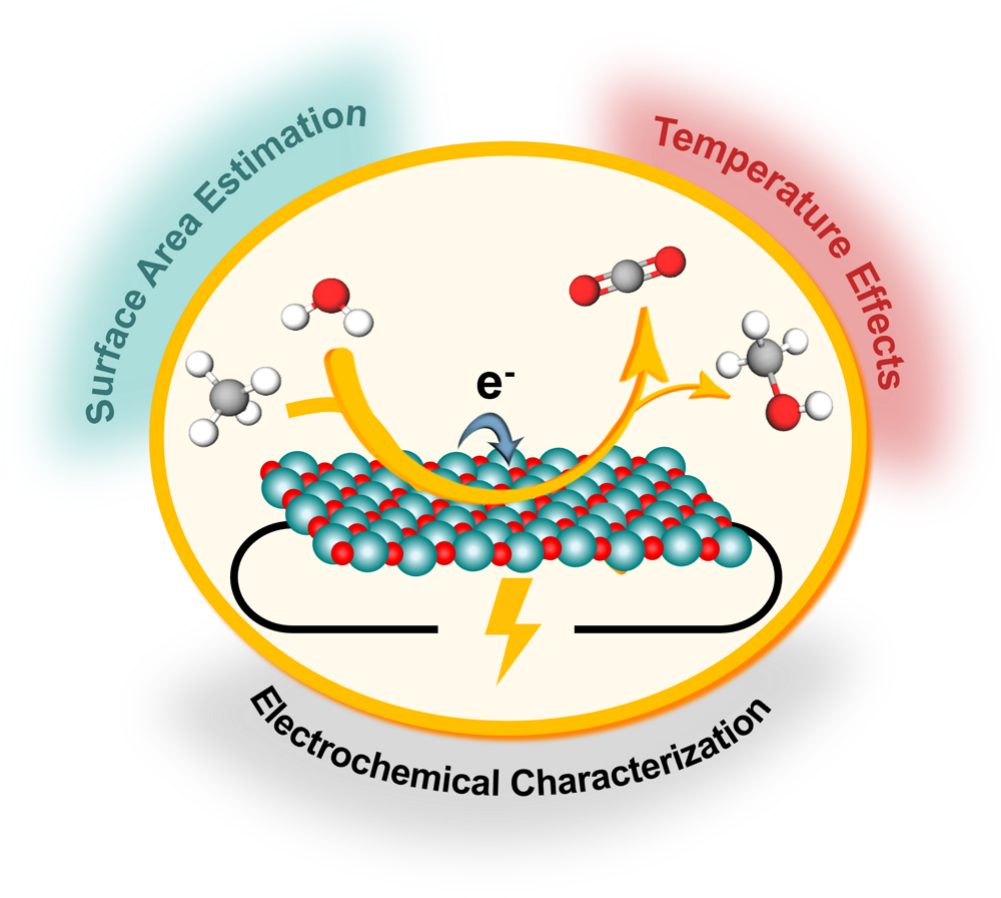
Representation of the main reactions encountered in electrochemical methane oxidation on metal oxides and summary of key parameters influencing the reaction activity and selectivity investigated in this work.

The partial methane oxidation to methanol on metal oxides requires reactive oxygenated species derived from water oxidation.^6,12,14^ According to theoretical calculations,^15^ water oxidation, or the oxygen evolution reaction (OER), starts with the formation of an adsorbed hydroxide group on the metal oxide (reaction 1), which then undergoes dehydrogenation to produce an adsorbed oxygen atom O* (reaction 2), followed by the formation of a peroxide intermediate (reaction 3), and finally the desorption of oxygen (reaction 4).

1 *+H2O→O*H+H++e−

2 O*H→O*+H++e−

3 O*+H2O→O*OH+H++e−

4 O*OH→*+O2+H++e−

For the case where reaction 3 is the potential-determining step, there will be a potential window where *O intermediates are stable. Theoretical calculations by Arnason et al. on some metal oxides suggest that these stable *O species, instead of proceeding through the typical oxygen evolution pathway (reactions 3 and 4), they can thermally activate methane (reaction 5), leading to the formation of *O···CH_4_. According to DFT simulations, this step can then be followed by the formation and desorption of methanol (reactions 6 and 7).^8^

5 O*+CH4→O*···CH4

6 O*···CH4→O*H···CH3

7 O*H···CH3→CH3OH+*

For this pathway to be viable, step 3 must be the potential-determining step; otherwise, the formation of a stable *O species would not be possible. This limits the selection of methane oxidation catalysts to those with strong enough *O binding energy that the free energy of reaction 3 (*G*_HOO*_ – *G*_O*_) is higher than that of reaction 2 (*G*_O*_ – *G*_HO*_), introducing the first requirement that Δ*E*_3_ – Δ*E*_2_ > 0.^8^ At the same time, weak *O bonds are necessary to activate methane at reasonable rates (reaction 5). This introduces competing requirements and identifies catalysts lying at the top of the OER volcano (such as IrO_*x*_ and RuO_*x*_), as the most active for methanol formation. Moreover, for the preferential formation of methanol over oxygen, *G*_HOO*_ – *G*_O*_ (reaction 3) should also be higher than the methane activation energy (*E*_methane act_), introducing a second requirement that Δ*E*_3_ – *E*_methane act_ > 0. Arnarson et al. identified some metal oxides that might be selective for the electrochemical methane to methanol conversion according to the two criteria Δ*E*_3_ – Δ*E*_2_ > 0 and Δ*E*_3_ – *E*_methane act_ > 0.^8^

The values of Δ*E*_3_ – Δ*E*_2_ and Δ*E*_3_ – *E*_methane act_ are shown in Figure 2 for a selection of metal oxides, where IrO_2_, MnO_2_, RuO_2_, and VO_2_ are promising candidates.^8^ However, oxides like IrO_2_ and RuO_2_ are also active electrocatalysts for the oxygen evolution reaction in acidic media,^16–18^ and since water and methane oxidation typically occur at similar potentials on metal oxides, there only exist a narrow potential window to convert methane to methanol without competing with the OER.^8^ This poses an intrinsic limitation on both the selectivity and the current density that methane oxidation on metal oxides can achieve. Nevertheless, these calculations suggest that metal oxides may be promising candidates for selective methane to methanol conversion.

**Figure 2:**
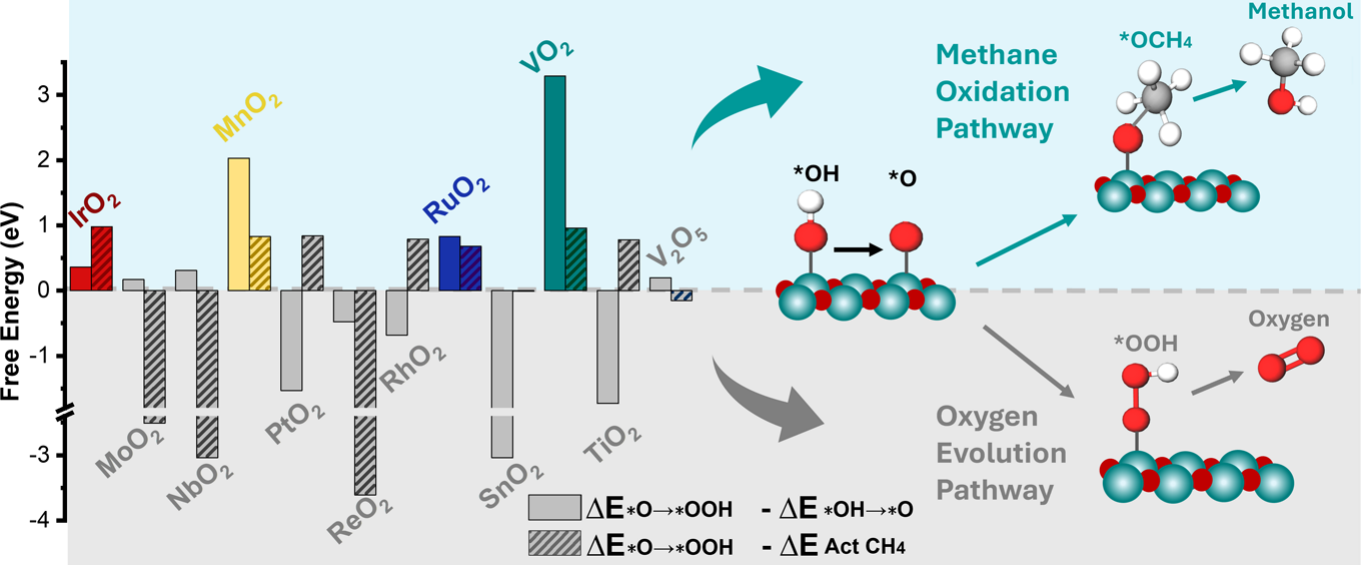
Difference between the free energy of steps 3 (*O to *OOH oxidation) and 2 (*O formation from *OH) for OER (solid bars) and step 3 of OER and methane activation (dashed bars). Data was obtained from Arnarson et al.,^8^ and the plot is adapted from Fornaciari et al.^7^ More data regarding the free energy of each step are shown in Figure S1.

However, while things might seem clear from a theoretical perspective, there are very few reliable experimental studies on monometallic metal oxides for methane electrooxidation. Prajapati et al. screened 12 transition metal oxides for methane oxidation, out of which all produced oxygen as the main product, none produced methanol, and only three (TiO_2_, IrO_2_, PbO_2_) were able to oxidize methane to CO_2_, in a neutral pH phosphate buffer.^11^ Later on, Shen et al. reported methane oxidation to methanol and acetate on NiO_*x*_, CoO_*x*_, CuO_*x*_, and MnO_*x*_ in potassium carbonate and potassium perchlorate, by reducing the applied potential, and changing the testing configuration to a rotating cylinder.^19^ Although they did not calculate the selectivity and the production was limited to around 30 μM, these isolated reports already highlight the difficulty in comparing literature results, due to the different testing conditions and their overlooked effect on the reaction.^20–23^ An interesting and largely unexplored approach to increasing selectivity is through rational electrolyte^24^ or surface engineering.^25^ For the case of electrolyte engineering, it is worth mentioning a very recent work by Kim et al.,^24^ which was published while this manuscript was under revision. Kim et al. obtained a Faradaic efficiency of up to 70% for the production of methanol, using IrO_2_ as a catalyst. Compared to the work of Prajapati et al., who reported less than 10% Faradaic efficiency toward methane oxidation, and CO_2_ as the only product; the work of Kim et al. differs for the applied potential (1.5 V compared to 2.3 V vs RHE) and instead of an alkaline electrolyte, they used carbonate, which has been reported to form active *O species at lower potential than water.^26^ This highlights the fact that methane oxidation activity and selectivity does not only depend on the catalyst of choice but is heavily dependent on the testing conditions.

Another important challenge for the study of the electrochemical methane oxidation is the lack of activity and selectivity descriptors. For more well-studied reactions, such as oxygen reduction (ORR) and evolution (OER), the establishment of activity descriptors^21,22^ has helped to rationalize the performance trends.^27^ For the case of ORR and OER, the discovery of linear scaling relationships among the binding energies of oxygenated intermediates made it possible to obtain volcano plots of the activity as a function of the binding energy of any intermediate. Elucidating the descriptors becomes challenging for reaction involving multiple pathways, leading to various products, and with reaction intermediates that can exhibit different scaling relationships. The combined effort of experimental and theoretical researchers has allowed the identification of key intermediates, the binding energy of which can act as descriptors to understand the selectivity trends for CO2 reduction.^28,29^

In the past five years, model studies have been carried out on Pt catalysts for electrochemical methane activation and oxidation. These works have shown that the adsorption of methane is the rate-determining step, and that it is weakly potential-dependent.^30–32^ These works have set a first step toward establishing a protocol for electrochemical methane oxidation, composed by a potential hold, where methane can be thermally chemisorbed and electrochemical oxidized to *CO, followed by a potential sweep where *CO is oxidized to CO_2_.^31,32^ Interestingly, fundamental investigations on Pt single-crystalline surfaces show strong structure sensitivity for methane oxidation. Although, to date, methane oxidation on platinum yields only CO_2_, Pt remains an interesting system for studying the fundamental mechanisms of methane adsorption and electrooxidation. In contrast, little has been established for metal oxides, for which no reliable testing protocol has yet been developed, and the reaction mechanism remains poorly understood.

Activity or selectivity descriptors for methane electrooxidation on metal oxides have yet to be identified, and the influence of key experimental parameters remains unexplored. Furthermore, there is no standardized protocol for testing methane oxidation catalysts, and no consensus on how to report selectivity. In most cases, the detailed experimental protocol is not even reported, making it extremely challenging to compare literature results and evaluate the impact of testing methods. The same challenges are being faced for the detection of methane oxidation products, for which systematic investigations and reliable protocols for quantitative product detection are also lacking. However, in this work, we focus on reliably establishing the electrochemical performance and do not address product detection, which remains an important direction for future research in the field.^13,34^

In this work, we propose a testing protocol for the reliable and reproducible measurements of methane electrooxidation, using IrO_*x*_ as a model electrocatalyst, and we apply this protocol to investigate the effect of various experimental parameters.^13^ We use carefully designed experiments to show the limitations of our current understanding of methane electrooxidation mechanism, through the investigation of three experimental parameters: (i) surface area estimation, (ii) temperature, and (iii) electrochemical characterization technique and testing protocol (Figure 1). These parameters have been largely overlooked in the literature for electrochemical methane conversion, despite reports of their strong effects on other electrochemical reactions.

i) The first parameter is the choice of normalization for the current density, which will be assessed by comparing the results normalized by geometric surface area and the electrochemically active surface area. This will show the limitation of normalization by the geometric surface area, which is still the most adopted in methane electrooxidation thus far.^4,21,22,35–37^

ii) Second, we will discuss the effect of temperature by carrying out experiments at 25 and 50 °C. While temperature always affects the rate of electrochemical reactions, this parameter might be critical in methane oxidation on metal oxides, where activation of methane (reaction 5) has been suggested to be thermochemical.^8^
iii) Finally, we will address the importance of the electrochemical technique, by comparing the results obtained via cyclic voltammetry and chronoamperometry, and we will propose a testing protocol for the acquisition of reproducible results.

To date, neutral,^33,34^ alkaline,^13,35^ carbonate-based,^13,19,24^ and nonaqueous^36^ electrolytes have been reported for the electrooxidation of methane on metal oxides. The scattered experimental data available suggest that the choice of electrolyte is likely to influence the activity and selectivity of methane electrooxidation, but the exact effect is not understood due to the lack of systematic studies. Therefore, we attempt to use an electrolyte that would have minimal interaction with the catalyst. The choice was 0.1 M HClO_4_, which was identified as the least disruptive electrolyte in our previous work on IrO_*x*_ nanoparticles for the OER.^37^

Moreover, to obtain reliable and reproducible measurement of the electrochemical methane oxidation reaction activity, we: (i) started each measurement from a consistent initial state of the catalyst surface, by following a rigorous testing protocol, and (ii) accounted for any changes in the initial catalyst structure, by normalizing the current by the electrochemically oxidized surface area (EOSA). Even when care is taken to ensure a reproducible pretreatment of the catalyst, the amount of available IrO_*x*_ site can change for the same geometric area due to potential-induced restructuring of the surface. Figure 3a shows a schematic illustration of the polished iridium surface (on the left), and of the Ir/IrOx surface after the electrochemical oxidation protocol. The geometric surface area (shown in green), is commonly used to normalize the current, obtaining the geometric current density, but this normalization technique can mask changes in the intrinsic activity. Thus, we normalized the results by the electrochemically oxidized surface area (shown in blue), which provides estimate of the electrochemically active IrO_*x*_ sites, and is used to calculate the molar activity.

**Figure 3:**
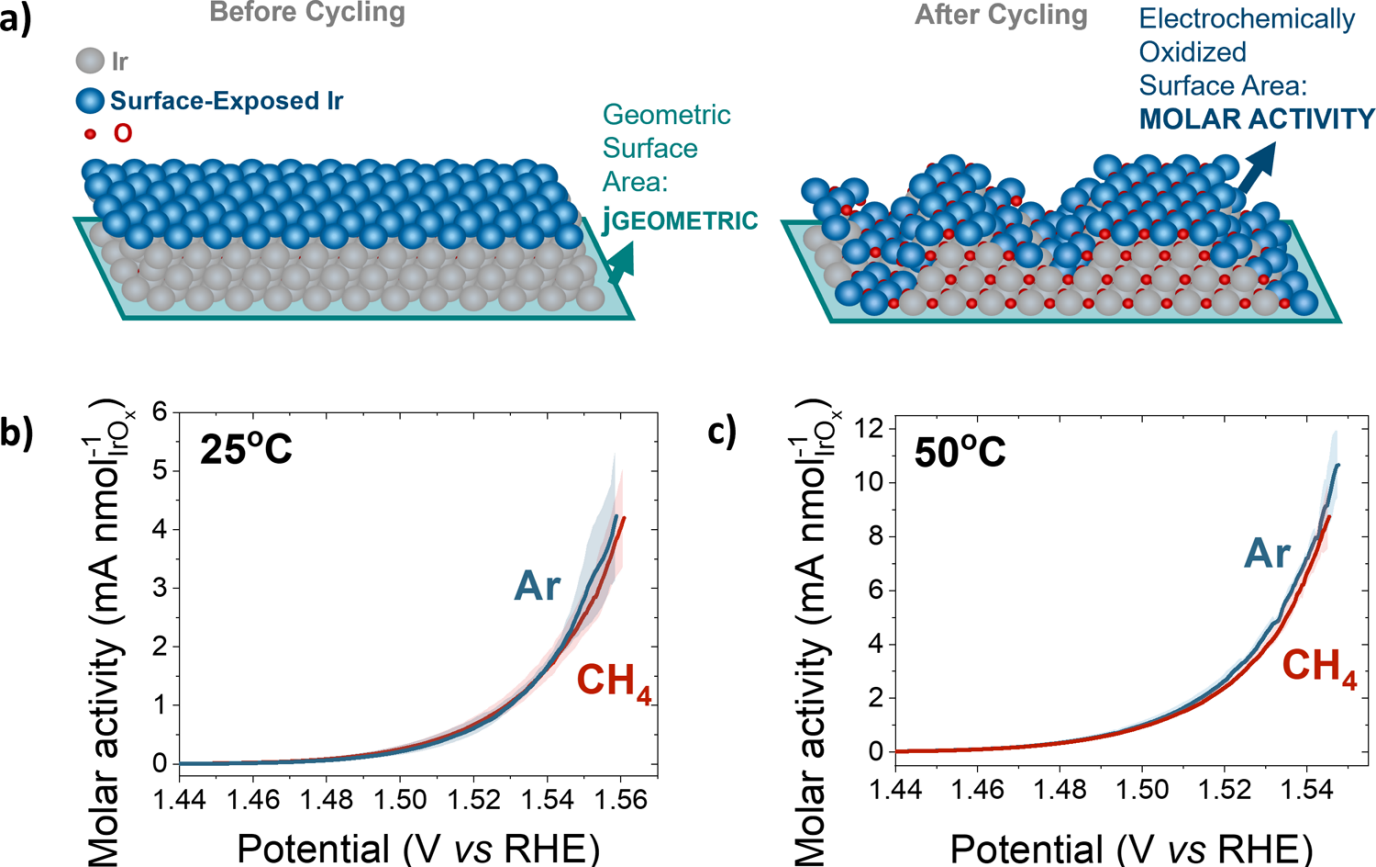
(a) Schematic representation of the geometric and electrochemically oxidized surface area (EOSA), before and after cycling. (b, c) Cyclic voltammograms of polycrystalline Ir-IrO_*x*_ in argon-saturated (blue) and methane-saturated (red) 0.1 M HClO_4_ at 1600 rpm and 5 mV s^–1^. The results in (b) and (c) were obtained at 25 and 50 °C, respectively, and are normalized by the EOSA. The shaded areas of each curve represent the standard deviation.

In a typical measurement, first a polycrystalline Ir disc was polished, cleaned and electrochemically oxidized by performing 100 cyclic voltammetry cycles at 100 mVs^–1^, between 0.025 and 1.40 V vs RHE in Ar-saturated electrolyte, attaining an oxidized surface of iridium and iridium oxide (Ir-IrO_*x*_) (see SI for further details, and Figure S4 for a schematic of the protocol). The last cycle was used to determine the EOSA by integrating the reversible Ir^III^/Ir^IV^ redox peaks before each measurement (further details are explained in the SI).^38^ After electrochemically oxidizing the surface, we saturated the electrolyte with the desired gas, we applied the desired potential, and we performed CVs or CAs in the OER region (limiting the upper potential to 1.6 V vs RHE to avoid the formation of soluble Ir species^8^). After this, the EOSA was calculated again to assess changes in the surface. To then perform a different measurement (changing temperature, gas or testing technique), the protocol was repeated from the beginning, starting from a freshly polished and oxidized IrO_*x*_ surface, and determining a new EOSA. This approach ensured that the initial state of the catalyst remained the same between each experiment, and that any restructuring of the surface (which is especially critical at high potential and in acidic media) is accounted for by determining the EOSA. Moreover, it allowed us to obtain reproducible results and consistent trends. To confirm this, each measurement was repeated three times, and we report the average of these measurements along with the standard deviation. While the importance of normalizing the current by electrochemically accessible sites and showing repeats is not unique to methane oxidation, it is specially critical here because of the close onset potential for water and methane oxidation, and the resulting small differences in current. In absence of reliable and reproducible protocols, catalyst degradation or experimental errors can be misinterpreted for changes caused by the presence of methane. In our experience, switching gases during the same experiment, or conducting various measurements without restarting from a fresh surface yielded such irreproducible and randomized changes, while the protocol described here always yielded consistent results. This is especially critical if we consider that most of the literature on methane oxidation does not specify which experiments were performed on a “fresh” surface or in what order the measurements were conducted. Moreover, repeats and the standard deviation between them are almost never reported.

Figure 3b,c shows the cyclic voltammograms of IrO_*x*_, in the oxygen evolution region (between 1.15 and 1.6 V vs RHE) under argon- and methane-saturated 0.1 M HClO_4_, obtained at 25 and 50 °C, and normalized by the EOSA (molar activity).^8^ The CVs were performed at a low scan rate of 5 mV s^–1^, under the assumption that methane adsorbs slowly on the surface as it has been observed in the case of metal surfaces, like Pt.^8,31,32,42^ Our results at 25 °C (Figure 3b) show that the molar activity is the same in argon and methane electrolytes, preventing any conclusive deduction regarding the effect of methane addition on the reaction and even suggesting that IrO_*x*_ might not be active for methane oxidation.

We then investigated temperature effects on electrochemical methane activation and oxidation on IrO_*x*_, following the DFT-motivated suggestion that methane activation might proceed through a thermochemical step.^8,32^ Under this assumption, one would expect that a mild increase in temperature might favor the methane oxidation reaction over the OER.^8^ Our results at 50 °C (Figure 3c) do indeed show an increase of current density with temperature, but the activity enhancement appears equivalent in argon- and methane-saturated electrolyte, and once again the two curves show minimal differences between each other. Therefore, from the CV data collected so far, not much can be concluded regarding the effect of methane.

Interestingly, a completely different picture appears when the reaction is measured using chronoamperometry, as shown by the results in Figure 4. Each measurement was performed for 15 min at potential values between 1.5 and 1.6 V vs RHE, in argon- and methane-saturated electrolytes, at 25 and 50 °C (more data at intermediate potentials can be found in the SI, Figure S7). The current density was normalized to the EOSA, which was once again obtained before each measurement, by integrating the reversible Ir^III^/Ir^IV^ redox peaks. Each measurement was repeated three times, and the average current density is shown here, with the shaded area representing the standard deviation.

**Figure 4:**
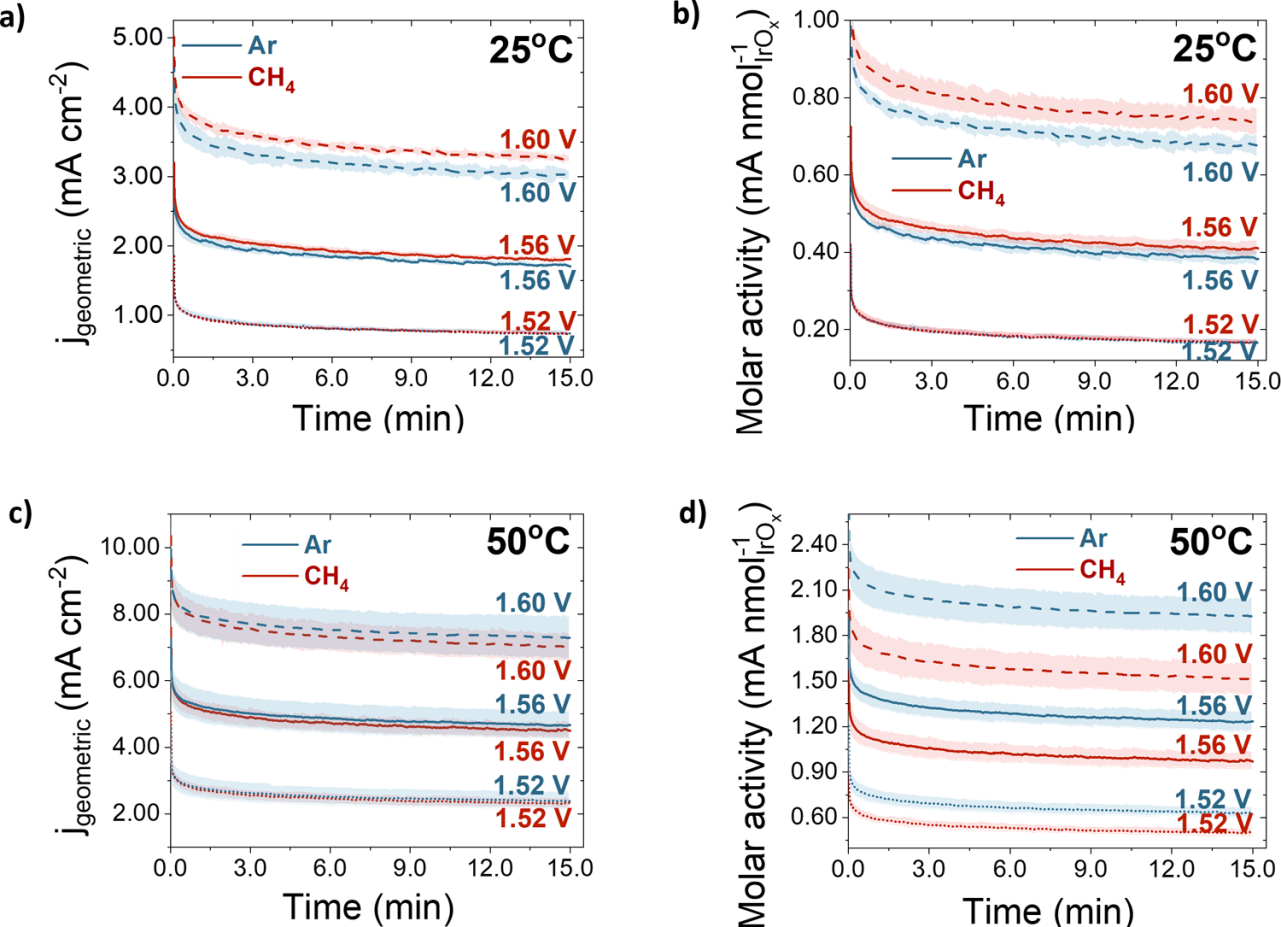
Chronoamperometry experiments in argon-saturated (blue) and methane-saturated (red) 0.1 M HClO_4_ at 1600 rpm, obtained at (a) 25 °C, normalized by the geometric surface area, (b) 25 °C, normalized by the electrochemically oxidized surface area (EOSA), (c) 50 °C, normalized by the geometric surface area, and (d) 50 °C, normalized by the EOSA. Each line represents data collected at different potential (1.52, 1.56, and 1.60 V vs RHE). Each measurement was repeated three times, and the average current density is shown, with a shaded area representing the standard deviation.

The first thing that we would like to highlight is the drastic difference between cyclic voltammetry and chronoamperometry measurements. While the CVs display no appreciable difference between the current recorded in the presence or absence of methane, CA experiments show clear activity enhancement in methane-saturated electrolyte at 25 °C (Figure 4a,b), and the opposite trend at 50 °C (Figure 4c,d). These differences are present in the entire potential range tested, even though they are more pronounced at higher potentials. Noticeably, these differences between CV and CA results mean that cyclic voltammetry alone is not a suitable technique to assess the methane electrooxidation activity of metal oxides, even as a first-instance screening tool. However, CVs are still used to screen catalysts for methane oxidation, or to draw conclusions regarding the reaction, in absence of other electrochemical data. For example, several reports claim to observe methane adsorption solely based on the CV, or only present electrochemical results from cyclic voltammetry.^24,39–42^

Second, it is worth noticing the difference between the results normalized by the geometric and the electrochemically oxidized surface area in Figure 4. While the two show similar trends, the difference between the geometric current density in argon- and methane-saturated electrolytes is barely visible, while the trends become much more obvious once the current is normalized by the EOSA. As can be noticed, when the molar activity is plotted, every single repeat displays the same trend as evidenced by the fact that the curves in methane and argon, with their respective standard deviations, never overlap.

Our results in Figure 4 additionally show the drastic and unexpected effect of temperature. While the activity at 25 °C increases in the presence of methane, it decreases at 50 °C. The contradictory differences between CV and CA results, as well as the unexpected temperature dependence prompt us to reconsider the proposed mechanism for methane oxidation. According to the mechanism suggested from theoretical calculations,^8^ a small potential window exists where *O species are stable and can activate methane before oxygen evolution is observed. From an experimental perspective, this should translate to a slight decrease in the onset potential after the introduction of methane; however, this is not observed. Furthermore, the temperature dependence remains puzzling considering the proposed mechanism, as if the methane activation step was chemical, as suggested by the theoretical works,^8^ one would expect methane activation and oxidation to be enhanced at higher temperature, which does not explain the current reduction at 50 °C. Equally notable is the discrepancy between results obtained from cyclic voltammetry and chronoamperometry under otherwise identical conditions. These differences could be caused by multiple effects, but most importantly they cannot be explained by our current understanding of the reaction mechanism.

This prompts us to conclude that our current understanding of the reaction mechanism is at least incomplete, and that electrochemical techniques alone are insufficient to gain the level of mechanistic understanding necessary to drive the field forward. While electrochemical techniques can probe different reaction conditions and give us invaluable insights into the reaction pathway, we envision that it will be the combination of electrochemistry with *in situ* characterization which will provide insight into the reaction mechanism. We anticipate that *in situ* spectroscopic techniques, such as infrared and Raman spectroscopy, will be essential to this aim.

In conclusion, it appears clear that, even for a model system like iridium oxide, several parameters such as temperature, electrochemical protocols and surface area estimation can affect the results. Our measurements show the drastic effect of each of these parameters on the performance of electrochemical methane activation and oxidation, which cannot be explained by our current understanding of the reaction mechanism. Furthermore, the lack of understanding of exactly how and to what extent experimental parameters affect the performance makes it extremely challenging to compare results collected at different conditions. Therefore, we believe that significant progress in the field will require:

i) Standardized protocols for assessing and reporting methane electrooxidation measurements: With this aim, here we propose a testing protocol based on preparing a fresh catalyst surface, determining the EOSA before each measurement, and reporting results normalized in this way. We suggest that, as for other electrocatalytic reactions, it should be standard practice to report the exact order of each measurement and to provide at least three independent measurements, with standard deviations.
ii) Fundamental studies of the reaction mechanisms on model catalysts: We envision that this will require a combination of controlled, fully characterized, and detailed electrochemical experiments, along with *in situ* characterization techniques.
iii) Systematic investigation of the impact of key experimental parameters: Here, we have only highlighted the strong effect of three selected parameters: temperature, electrochemical characterization technique and normalization. However, the reasons why these parameters influence the activity so strongly remain unclear, and many other experimental parameters still need to be explored.

## Supplementary Material



## Notes

Additional experimental details, electrochemically oxidized surface area (EOSA) calculation details, description and schematic of the testing protocol, and additional electrochemical results (PDF)
