# Supplementary material for: Why Testing Protocols Matter in Electrochemical Methane Oxidation: Insights from IrO x in Acid
Source: ACS Energy Lett. 2025 Sep 8;10(10):4842–8. doi: 10.1021/acsenergylett.5c01848 (PMC12519492; doi:10.1021/acsenergylett.5c01848)
Supplement: Supplementary file 1 [file nz5c01848_si_001.pdf]

Supporting Information

Why Testing Protocols Matter in Electrochemical Methane Oxidation: Insights from IrO_x in Acid

José Alejandro Arminio-Ravelo,[#,a] Silvia Favero, [#,b] María Escudero-Escribano[*,b,c]

[a] *Department of Chemistry, University of Copenhagen, Universitetsparken 5, 2100 Copenhagen (Denmark).*

[b] *Catalan Institute of Nanoscience and Nanotechnology (ICN2), Edifici ICN2, CSIC and Barcelona Institute of Science and Technology, UAB Campus, 08193 Bellaterra, Barcelona (Spain).*

[c] *Catalan Institution for Research and Advanced Studies (ICREA), Passeig de Lluís Companys, 23, 08010 Barcelona (Spain).*

**Corresponding author:*
E-mail: maria.escudero@icn2.cat

[#]*J.A.A.-R. and S.F. contributed equally to this work. These authors are co-first authors.*

Theoretical free energy plots for methane electro-oxidation

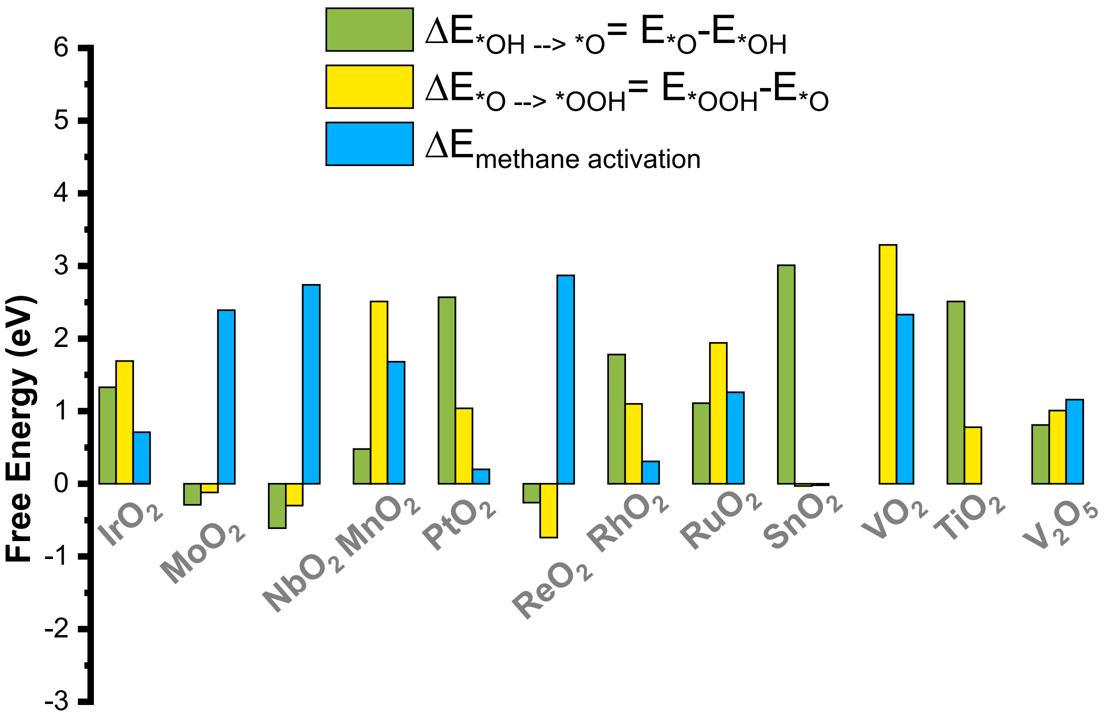

Figure S1: The free energies of various reaction steps are shown for a selection of metal oxides. The green bars indicate the energy barrier for the second step of oxygen evolution (OER) (*OH → *O + H^+ + e^-). The yellow bars represent the barrier for the third step of OER (*O + H_2O →*OOH + H^+ +e^-). The blue bars correspond to the is the energy barrier for methane activation (*O + CH_4 → *O···CH_4). These values were obtained from Arnarson et al.[1] and used to generate Figure 2 in the manuscript.

Experimental section

- **Electrochemical cell configuration**

For the electrochemical experiments, we used a borosilicate H-cell made by Leidse Instrument makers School in Leiden (Figure S1). The cell was divided into three compartments for each type of electrode: counter electrode (CE, left), working electrode (WE, center), and reference electrode (RE, right). The counter electrode and working electrode compartments were divided and connected with a Nafion membrane. The two compartments were held together with the help of a press designed for the cell. The WE compartment contained a jacket for temperature control. It was connected to a bath circulator model CD200F from Julabo. In addition, the central compartment contained a glass cap with a gas inlet connected to a bubbler with frit to enhance gas dissolution in the electrolyte. The cap also contained a gas outlet for gas sampling if desired. The reference electrode compartment was connected with the working electrode compartment with a frit to diminish cross-contamination from the WE to the RE. We used a Pt mesh (>95.95%, Junker Edelmetalle) as CE, an Ir disc of 1 cm of diameter (99.9%, Alfa Aesar) in a rotating ring disc (RDE) configuration from Radiometer Analytical as WE, and a calomel

electrode (SCE) (KCl sat., SCHOTT®) as the reference electrode. The experiments were performed using a potentiostat model VSP from Biologic.

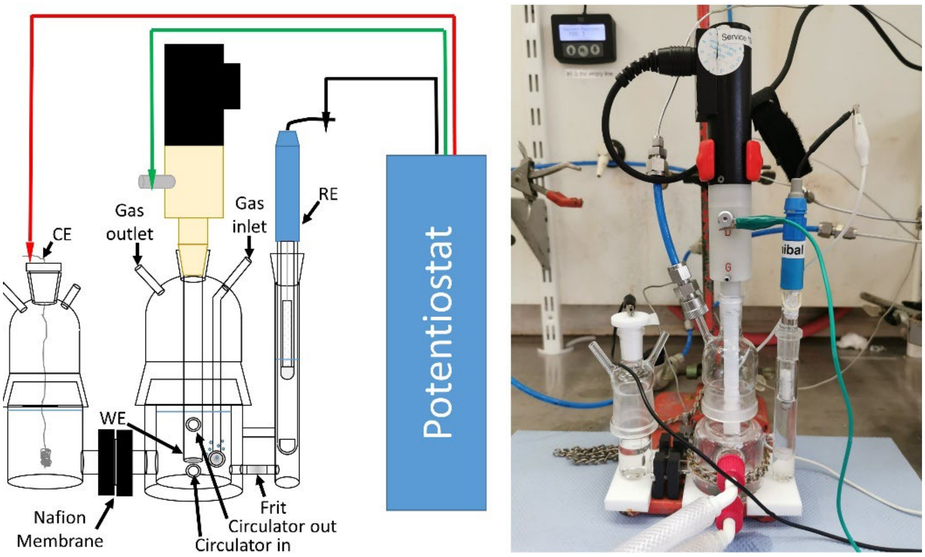

Figure S2. Schematic diagram (left) and photo (right) of the H-cell configuration.

- ## Cleaning and preparation of the electrochemical cell

The cleaning of the electrochemical cells was essential to avoid cross contamination between experiments. The H-cell was deeply cleaned as received. First, the components were immersed in a Piranha solution (3:1 concentrated H_2SO_4:H_2O_2) (H_2SO_4 $\geq$ 95%, Fisher Chemical; H_2O_2 30%, Fisher Chemical) for a least 12 h. The components were rinsed and boiled three times in Milli-Q® water. After the initial cleaning, between experiments, the H cell and its components were thoroughly rinsed multiple times with Milli-Q® water.

To activate the Nafion membrane that separates the working and counter electrode compartments, it was immersed in a 5 wt% H_2O_2 solution at 80 °C, then in Milli-Q® water at 80 °C, and lastly in 8 wt% H_2SO_4 solution at 80 ºC for 30 minutes each. Between immersions, the membrane was rinsed multiple times with Milli-Q® water. The membrane was also rinsed and kept in Milli-Q® water before and after the electrochemical experiments.

- ## Electrode preparation

The Ir polycrystalline electrode was polished at 500 rpm for 15 minutes using a polishing machine Labopol-20 brand Struers with a soft polishing cloth MD-Floc brand Struers, and an alumina suspension of 0.3 µm of particle diameter. Then, the disc electrode was rinsed three times with Milli-Q® water by using an ultrasonic cleaner Emmi®-30HC brand Emag in bath cycles of 5 minutes. The disc was polished again at 500 rpm for 15 minutes, but now with a soft polishing cloth MD-NAP brand Struers

and alumina suspension of 0.05 µm of particle diameter. The disc was rinsed in ethanol using the ultrasonic cleaner in a bath cycle of 5 minutes, and five more times in Milli-Q® water in bath cycles of 5 minutes.

- **Electrochemically oxidized surface area (EOSA)**

We used an Ir polycrystalline disc electrochemically oxidized to Ir-IrO_x as catalyst. The Ir disc was treated and characterized using cyclic voltammetry. We applied 100 cycles between 0.025 V and 1.40 V *vs* RHE with a scan rate of 100 mV s^{-1} and rotation speed of 1600 rpm in 0.1 M $HClO_4$ (Suprapur® 70%, Merk) saturated with Ar (99.9999%, Air Liquid) at 25 °C. Up to 85% of the resistance was compensated during the measurements. The resistance was measured before the cyclic voltammetry at 0.40 V vs RHE from 3.50 kHz to 500 mHz at 0 rpm. From the Nyquist plot obtained, the resistance corresponded to the real variable contribution when the imaginary variable was equal to 0.[2]

To quantify the amount of IrO_x generated, we calculated the electrochemically oxidized surface area (EOSA), representing the surface concentration of metal oxide over the electrode. The estimation of the EOSA on the Ir-IrO_x polycrystalline disc was based on the work reported by Lettenmier et. al.[2] For the calculations, we used the 100th cycle to estimate the average charge of the redox peaks of the couple Ir(IV)/Ir(III) (ca. 0.93 V vs. RHE), as shown in Figure S2.

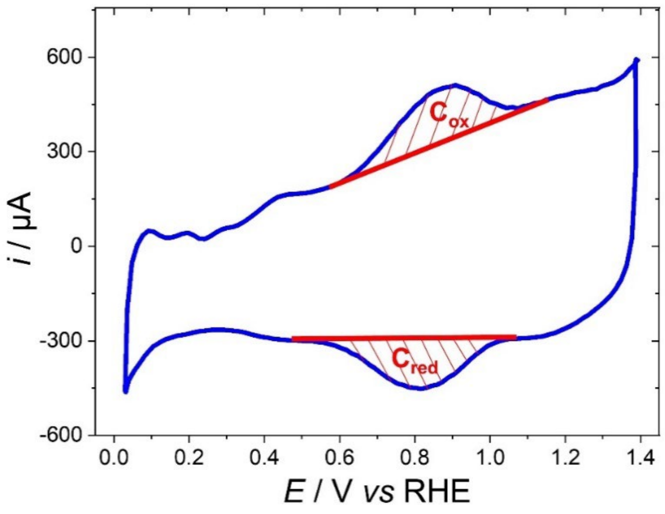

Figure S3. Cyclic voltammogram showing the charge integration of the Ir(IV)/Ir(III) pair on Ir-IrO_x polycrystalline disc.

Equation 1 was used to calculate the electrochemically oxidized surface area (EOSA), where C_{ox} and C_{red} are the charges passed through the oxidation and reduction of the Ir(III) / Ir(IV) pair respectively, F is the Faraday's constant (99485 C mol^{-1}), and A is the geometric area of the electrode. The calculation assumes that per electron transfer, one atom of Ir is oxidized or reduced.[3]

$$EOSA = \frac{C_{ox}+C_{red}}{2FA} \qquad (1)$$

The estimation of the EOSA also provided a quantitative parameter to evaluate the reproducibility of the electrode treatment between experiments. It was measured before each individual repetition of the cyclic voltammetry and chronoamperometry measurements at different gases and temperatures.

- **Cyclic voltammetry and chronoamperometry measurements**

The catalytic activity in the oxygen evolution region was evaluated using cyclic voltammetry between 1.00 and 1.60 V vs RHE with a scan rate of 5 mV s^{-1} and a rotation speed of 1600 rpm in 0.1 M $HClO_4$ (Suprapur® 70%, Merk). The electrolyte was saturated with Ar (99.9999%, Air Liquid) or CH_4 (99.9995%, AGA), and the temperature was 25 °C or 50 ºC. To ensure reproducibility, we performed each experiment at each gas and temperature three times.

The chronoamperometry measurements were carried out holding the potential for 15 minutes at 1.50, 1.52, 1.54, 1,56, 1.58, and 1.60 V *vs* RHE in progressive order with a rotation speed of 1600 rpm in 0.1 M $HClO_4$ (Suprapur® 70 %, Merk). The electrolyte was saturated with Ar (99.9999%, Air Liquid) or CH_4 (99.9995%, AGA), and a temperature of 25 ºC or 50 ºC. To ensure reproducibility, we performed each experiment at each gas and temperature three times.

- **Molar activity calculation**

To quantify the catalytic activity normalized by the number of active sites we calculated the molar activity. The molar activity was calculated using equation 2. The normalization assumed that IrO_x were the active sites for methane activation.

$$Molar\ activity = \frac{j_{geometric}}{EOSA} \qquad (2)$$

- **Testing Protocol**

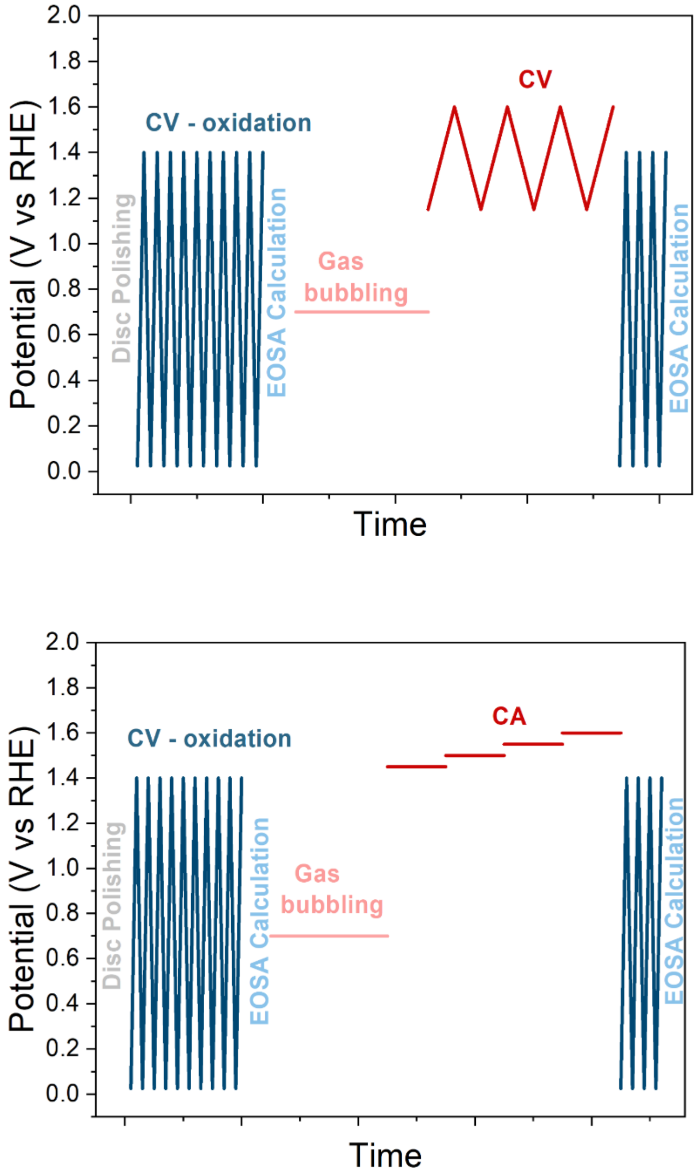

Figure S4. Schematic representation of the testing protocol used in this work.

For each CV or CA measurement described above, the steps described in Figure S3 were taken: the disc was polished and the cell cleaned as described above, then the Ir electrode was oxidized with 100 CV cycles and the EOSA was determined. The CV or CA measurement was performed, and then the EOSA was determined again at the end of three CV cycles. For each repeat, as well as for every measurement conducted at different temperatures or with a different gas, the protocol was restarted from the beginning.

Additional results:

- **EOSA measurements before the catalytic activity evaluation with cyclic voltammetry in the OER region.**

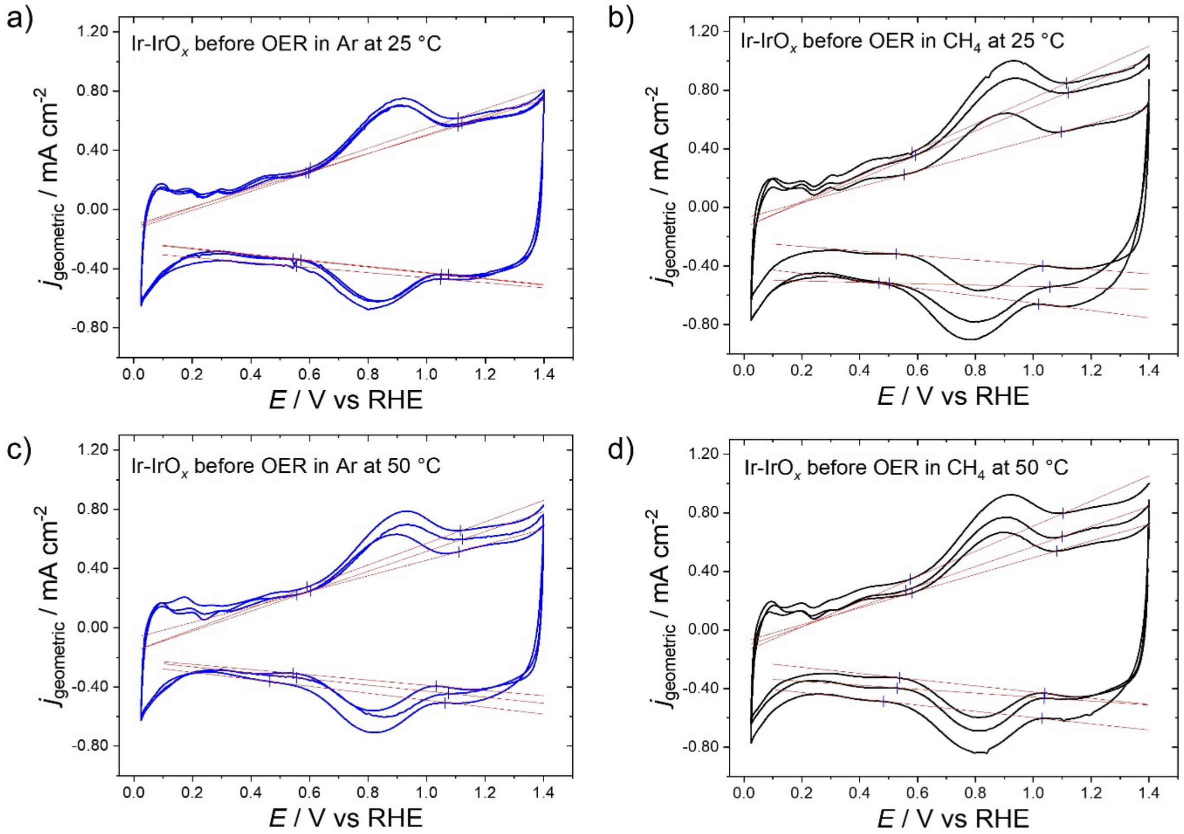

Figure S5. 100th-cycle cyclic voltammograms of the electrochemical oxidation of the Ir polycrystalline disc surface before each repetition of the activity evaluation in the oxygen evolution region in **a)** Ar at 25 °C, **b)** CH_4 at 25 °C, **c)** Ar at 50 °C**,** and **d)** CH_4 at 50 °C. Conditions: 1.00 to 1.60 V vs RHE with a scan rate of 5 mV s^{-1} and a rotation speed of 1600 rpm in 0.1 M $HClO_4$. The red lines indicate the baseline used as integration.

Table S1. Table summarizing the EOSA values measured before each activity evaluation with cyclic voltammetry in the oxygen evolution region.

Cyclic voltammetry in the oxygen evolution region	EOSA ($nmol_{IrO_x}$ cm^{-2})
Ar 25°C (1)	4.90
Ar 25°C (2)	5.04
Ar 25°C (3)	5.14
CH_4 25°C (1)	4.48
CH_4 25°C (2)	6.76
CH_4 25°C (3)	5.54
Ar 50°C (1)	5.70
Ar 50°C (2)	4.46
Ar 50°C (3)	4.56
CH_4 50°C (1)	5.00
CH_4 50°C (2)	6.04
CH_4 50°C (3)	4.56

- **EOSA measurements before the catalytic activity evaluation with cyclic voltammetry in the OER region**

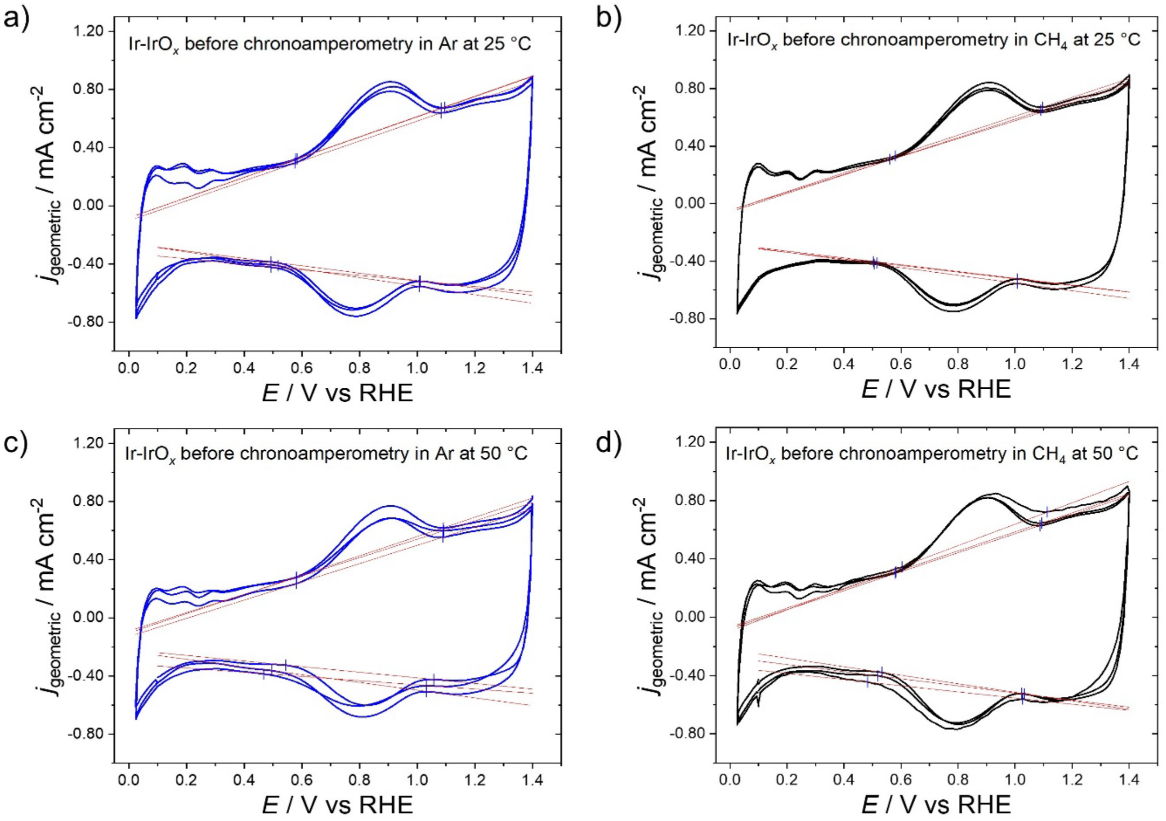

Figure S6. 100th-cycle cyclic voltammograms of the electrochemical oxidation of the Ir polycrystalline disc surface before each repetition of chronoamperometry measurements in **a)** Ar at 25 °C, **b)** CH_4 at 25 °C, **c)** Ar at 50 °C, and **d)** CH_4 at 50 °C. Conditions: 1.00 to 1.60 V vs RHE with a scan rate of 5 mV s^{-1} and a rotation speed of 1600 rpm in 0.1 M $HClO_4$. The red lines indicate the baseline used as integration.

Table S2. Table summarizing the EOSA values measured before each chronoamperometry experiment.

**Chronoamperometry experiment**	**EOSA ($nmol_{IrO_x}$ cm^{-2})**
Ar 25°C (1)	5.51
Ar 25°C (2)	6.09
Ar 25°C (3)	5.49
CH_4 25°C (1)	5.41
CH_4 25°C (2)	5.54
CH_4 25°C (3)	5.92
Ar 50°C (1)	5.44
Ar 50°C (2)	4.89
Ar 50°C (3)	4.19
CH_4 50°C (1)	5.75
CH_4 50°C (2)	6.13
CH_4 50°C (3)	5.83

- **Chronoamperometry measurements:**

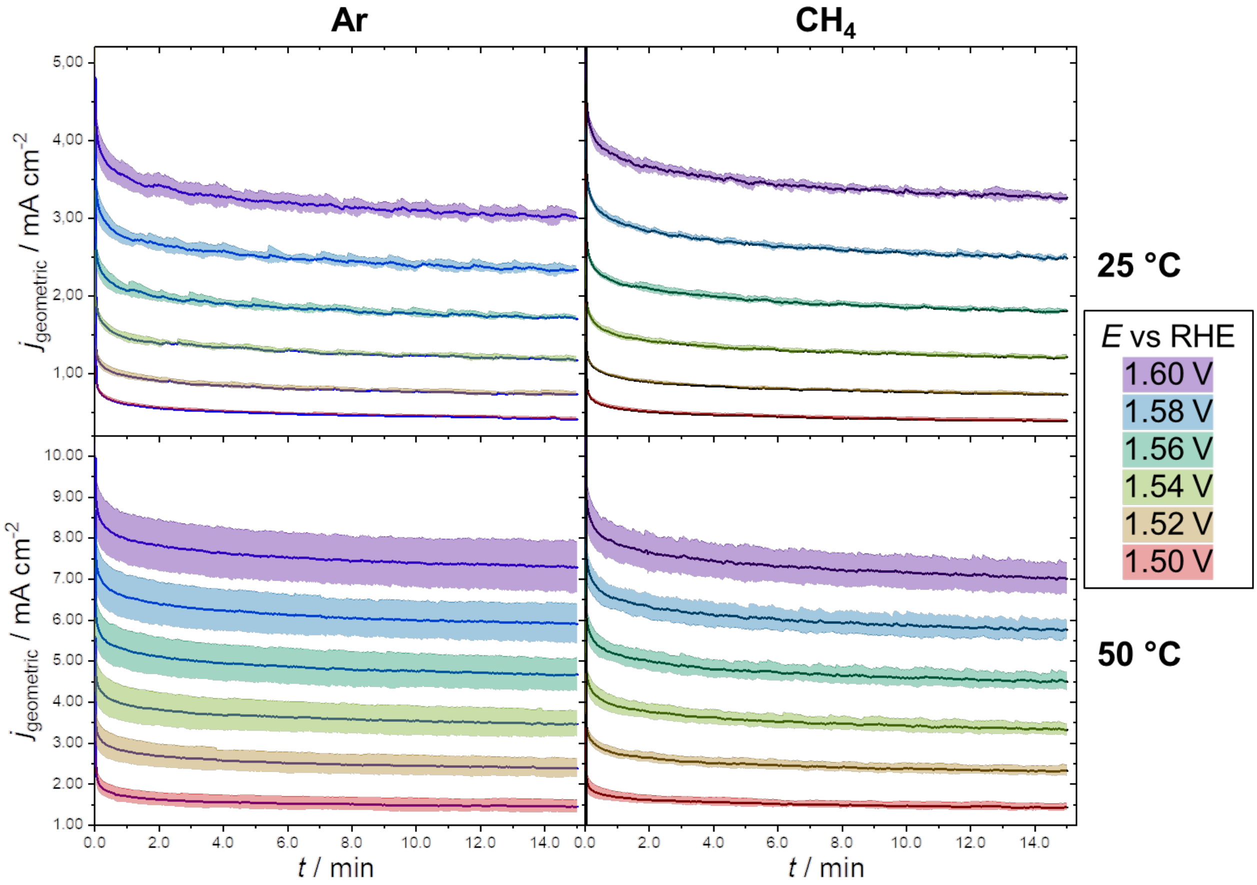

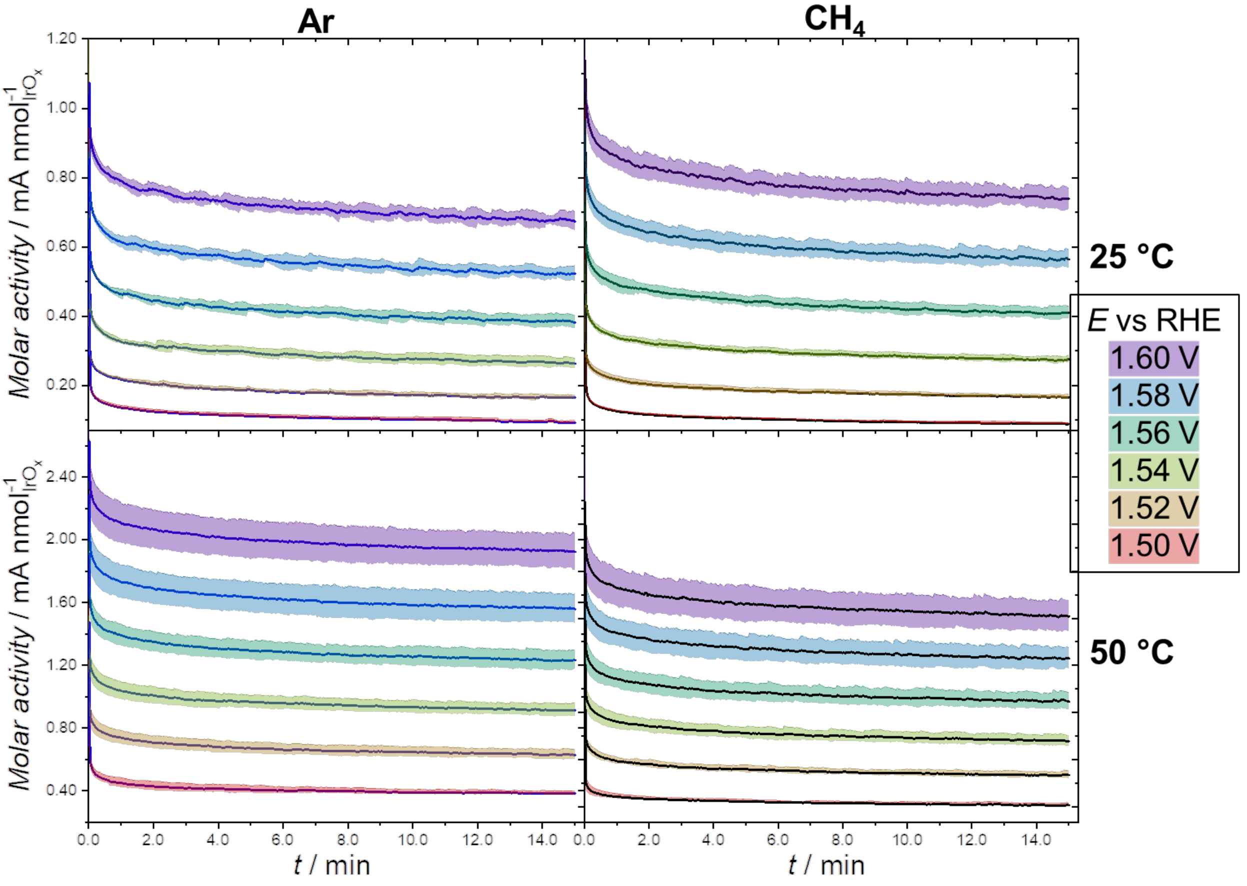

Figure S7. Chronoamperometry experiments at 25 °C and at 50 °C in Ar- and CH_4-saturated 0.1 M $HClO_4$. Each line represents data collected at different potential, according to the legend. The colored shadows represent the standard deviation of each measurement. All the results have been normalized by the geometric area ($j_{geometric}$) in the top figures and the EOSA (molar activity) in the bottom figures.

References

1. Arnarson, L.; Schmidt, P. S.; Pandey,M.; Bagger, A.; Thygesen, K. 508 S.; Stephens, I. E. L.; Rossmeisl, J. Fundamental Limitation of 509 Electrocatalytic Methane Conversion to Methanol. *Phys. Chem.* 510 *Chem. Phys.* 2018, *20* (16), 11152−11159.

2. Pletcher, D. *A First Course in Electrode Processes*. (The Royal Society of Chemistry, Cambridge, 2009).

3. Lettenmeier, P. *et al.* Nanosized IrO x -Ir Catalyst with Relevant Activity for Anodes of Proton Exchange Membrane Electrolysis Produced by a Cost-Effective Procedure. *Angewandte Chemie International Edition* 55, 742–746 (2016).
